# Carotid wall structure during and after 6 months of spaceflight

**DOI:** 10.3389/fphys.2025.1638531

**Published:** 2025-10-09

**Authors:** Philippe Arbeille, Kathryn Zuj, Danielle Greaves, Richard Hughson

**Affiliations:** ^1^ CERCOM-UMPS Faculte de Medecine (Université de Tours – France), Tours, France; ^2^ Faculty of Health, University of Waterloo, Waterloo, ON, Canada; ^3^ Schlegel-UW Research Institute for Aging, Waterloo, ON, Canada

**Keywords:** carotid, vessel wall, intima media thickness, index reflectivity, ISS, ultrasound

## Abstract

**Purpose:**

Long-duration spaceflight has been shown to result in vascular adaptation. However, little is known about the recovery of these parameters after the flight. The purpose of our study was to assess the common carotid (CC) artery wall properties before, during, and after 6 months of spaceflight on the International Space Station (ISS).

**Methods:**

CC artery wall properties were assessed using ultrasound measures of the intima media thickness (IMT) and the evaluation of the radiofrequency signal to determine the index of reflectivity (IR) for the posterior wall, posterior intima, and neck muscle. Data were collected from ten astronauts preflight (PRE), on flight day 150 (FD150), and 4 days (R+4) and 6 months (R+6 m) postflight.

**Results:**

IMT increased from PRE (0.56 ± 0.09 mm) to FD150 (0.65 ± 0.11 mm) and R+4 (0.65 ± 0.08 mm), and returned to PRE levels on R+6 m (0.57 ± 0.12 mm). Posterior wall IR also increased from PRE (63% ± 5.5%) to FD150 (78% ± 7.8%) and R+4 (86% ± 4.4%), and returned to PRE levels on R+6 m (60% ± 23%). In contrast, both intima IR and neck muscle IR increased slightly during spaceflight but returned to preflight levels on R+4.

**Conclusion:**

Changes in CC posterior wall IMT and IR that persisted at R+4 but normalized at R+6 m suggest structural or content modifications of the vessel wall. In contrast, the early recovery of neck muscle and posterior wall intima IR at R+4 suggests a transient process that is possibly related to microgravity-induced fluid shifts.

## Introduction

Changes in the vascular structure and function have been previously observed with long-duration spaceflight. Increased common carotid (CC) arterial wall intima media thickness (IMT) and decreased CC artery wall distensibility have been observed after 6 months on the International Space Station (ISS) (Hughson et al., 2016; Arbeille et al., 2017a). Changes in the CC artery wall structure and/or content resulting in increased ultrasound reflectivity (index of reflectivity, IR) have been identified through the assessment of the ultrasound radiofrequency signal (RF) in astronauts 4 days after returning to Earth following 6 months on the ISS and after 5 days of dry immersion as a simulation of microgravity exposure (Greaves et al., 2021: Arbeille et al., 2021; Arbeille et al., 2022). Microgravity-induced fluid shifts toward the head have also resulted in increased jugular vein volume (Arbeille et al., 2015), potentially leading to an increased risk of thrombus formation (Marshall-Goebels et al., 2017), and increased intracranial venous blood velocity (Arbeille et al., 2021; Arbeille et al., 2017b) due to the compression of the vein by the brain tissue (Roberts et al., 2017).

Whereas many of the observed vascular adaptations to microgravity exposure may result from fluid shifts toward the head, similar adaptations have also been noted in ground-based confinement studies, such as MARS-500 and CELSS (Yuan et al., 2019), suggesting the involvement of other factors. Reduced physical activity and environmental stress associated with spaceflight may contribute to altered glucose and calcium metabolism (Hughson et al., 2016; Arbeille et al., 2017a), contributing to the observed changes in vascular properties. However, it is unknown if the observed changes in vascular properties are present during spaceflight or if they return to preflight levels 6 months after returning to Earth.

The purpose of the current study was to investigate the CC artery and the surrounding (muscle) structure during long-duration 6-month spaceflight and at 4 days and 6 months after returning to Earth. It was hypothesized that the CC IMT and IR and the neck muscle IR would adapt with increased thickness and reflectivity during spaceflight and that some of these changes would persist till 4 days after returning to Earth. It was also hypothesized that these changes would recover 6 months after astronauts return to normal daily activities in Earth’s gravity.

## Research design and methods

### Subjects

Ten astronauts (seven males and three females) participated in this study. The average age of the astronauts was 44 ± 3 years, with the average height of 177 ± 5 cm and body mass of 76 ± 11 kg. All study protocols were approved by the University of Waterloo Office of Research Ethics Committee, Johnson Space Center Committee for the Protection of Human Subjects, NASA Human Research Medical Review Board, the European Space Agency Medical Review Board, and the Japanese Space Agency Research Ethics Board (study protocol #Pro1222; NASA MPA116301606HR; FWA00019876) in accordance with the Declaration of Helsinki. Each participant was informed in detail about the experiment, including the ability to withdraw from the study at any time without penalty, and they gave informed consent before participating.

### Experimental protocol

Ultrasound assessments were conducted before spaceflight (PRE), on approximately day 150 of the 6-month flight (FD150), 4 days after returning to Earth (R+4), and 6 months after returning to Earth (R+6 m). Preflight and postflight assessments were conducted by a trained sonographer with the astronaut resting in a supine position. During the inflight session, the astronaut was free floating and was instructed only to hold the ultrasound probe in place as all probe orientation and ultrasound functions were teleoperated by a sonographer on the ground. Data were collected using the Sonoscanner-CNES echograph (Orcheo Lite, Sonoscanner, Paris, France) that is presently onboard the ISS and at the NASA Johnson Space Center ground facility for pre- and postflight data collection (Arbeille et al., 2018). It includes a 17 MHz high-resolution linear array probe, motorized to be teleoperated (tilted and rotated), along with the ultrasound function (3D, Doppler, RF) from the ground. During the inflight session, the same presets as preflight (gain, depth, etc.) were used for each ultrasound assessment.

**FIGURE 1:**
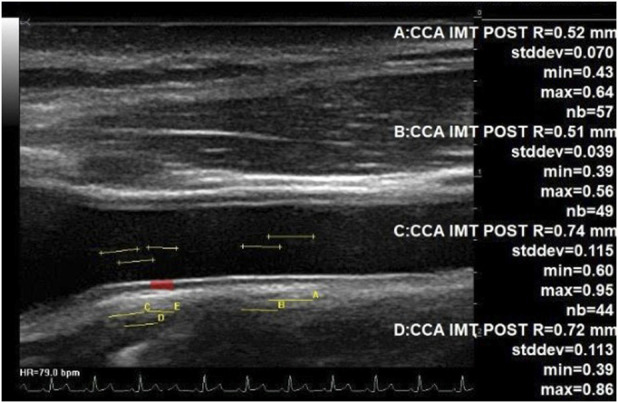
IMT measurement at the middle part of CC and at the bulb area. Echographic image of the common carotid artery up to the bulb area (beginning of bifurcation) at the left, showing automated assessment of the IMT. Each of the four measurements was completed on a total of 44 to 57 adjacent locations along the carotid posterior wall. The IMT changed from 0.51 mm (middle part of CC) up to 0.74 mm (beginning of the bifurcation).

For all assessments, the ultrasound probe was located on the right anterior surface of the neck, in a neck long-axis orientation, perpendicular to the skin and in contact with the collar bone, and then moved forward by some cm to the bifurcation. In both cases, the probe transducer was oriented by the expert on the ground until the CC artery posterior intima appeared clearly within the image. The IMT was then measured on this view at the carotid bulb area (beginning of the bifurcation) and in the middle of the image using an automated detection of the intima and vessel wall limits (Figure 1).

While maintaining the probe position used for the IMT assessments, radio frequency (RF) signal capture was initiated, lasting approximately 2 seconds. Using the amplitude of the captured signal (displayed in an Excel file), the IR was calculated (along six adjacent vertical lines selected on the carotid echographic image) as the quotient of the energy backscattered by each tissue of interest and the total energy returned to the ultrasound probe (Arbeille et al., 2021). The IR was determined for the CC artery posterior wall, posterior intima, and lumen in the middle of the artery image away from the carotid bulb, along with a measure of neck muscle IR. The same number of Excel cell along the RF line was used at each session.

### Statistical analysis

The effects of spaceflight and recovery were determined using a one-way repeated measures analysis of variance (SigmaPlot 12.5, Systat Software Inc., San Jose, CA). All the assessed variables passed tests for normality (Shapiro–Wilk test) and equal variance (Levene’s median test). In the case of significant main effects, Tukey *post hoc* testing was performed to test for statistical significance of pairwise comparisons. For all the tests, significance was set at p < 0.05, with data reported as the mean ± standard deviation.

## Results

**FIGURE 2:**
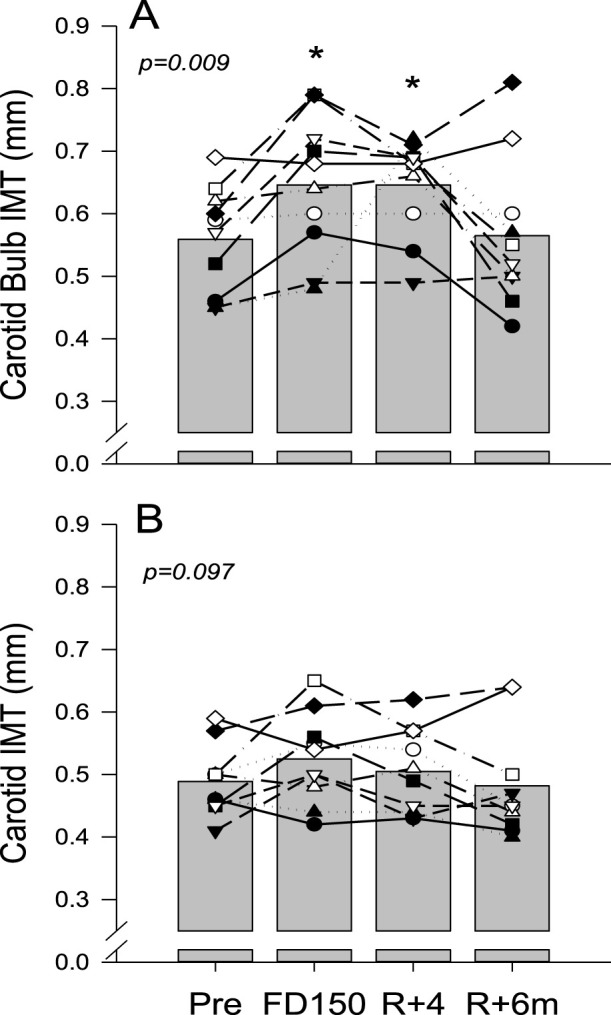
Mean (bars) and individual (symbols) measures of common carotid artery IMT measured at the bulb area away from the carotid bulb **(A)** and measured in the middle part of the artery, further away in the bulb area **(B)**. Data are shown for preflight (PRE), flight day 150 (FD150), 4 days after returning to Earth (R + 4), and 6 months after returning to Earth (R+6 m). The main effects of measurement time are indicated on the graphs with values that are different from those of PRE, indicated by p < 0.05. Lines connecting symbols are included to identify individual trends and do not represent changes in the variables over time.

Ultrasound assessments for CC artery IMT measurements were successfully conducted on all ten astronauts. However, due to technical issues, RF data were not collected for one of the astronauts. The CC artery posterior wall IMT in the carotid bulb area was found to be affected by spaceflight and recovery (Figure 2A; p = 0.009). During spaceflight, IMT increased from 0.56 ± 0.09 mm PRE to 0.65 ± 0.11 mm on FD150 (p = 0.048), remained elevated at 0.65 ± 0.08 mm on R+4 (p = 0.048), but returned to 0.57 ± 0.12 mm on R+6 m (p = 0.998). In contrast, although IMT measured further away from the carotid bulb (Figure 2B) appeared to increase with spaceflight (0.49 ± 0.06 mm PRE vs. 0.53 ± 0.07 mm on FD150), the effects of spaceflight and recovery were not statistically different (p = 0.097).

**FIGURE 3:**
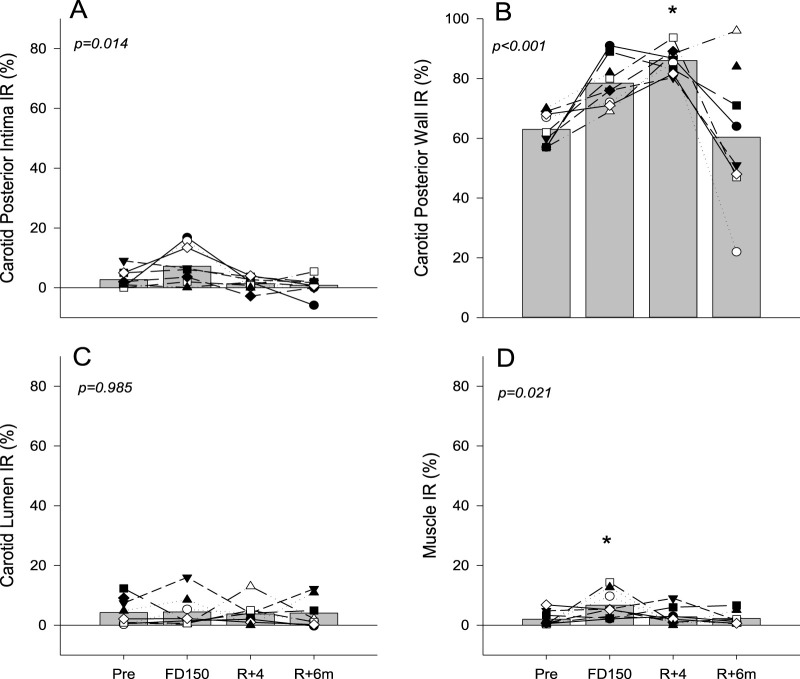
Mean (bars) and individual (symbols) index of reflectivity (IR) measures of the posterior wall intima **(A)**, posterior wall **(B)**, vessel lumen **(C)**, and neck muscle **(D)**. Data are shown for preflight (PRE), flight day 150 (FD150), 4 days after returning to Earth (R + 4), and 6 months after returning to Earth (R + 6 m). The main effects of measurement time are indicated on the graphs with values that are different from those of PRE, indicated by p < 0.05. Lines connecting symbols are included to identify individual trends and do not represent changes in the variables over time.

Analysis of the RF signal found that the posterior wall IR tended to increase on FD150 from PRE values (63% ± 5.5% PRE vs. 78% ± 7.8% FD150; p = 0.07), was significantly elevated on R+4 (86% ± 4.4%; p = 0.001), and returned to preflight levels on R+6 m (60% ± 23%; p = 0.979 Figure 3B). Neck muscle IR was elevated during spaceflight (2.0% ± 2.4% PRE vs. 6.7% ± 4.5% FD150; p = 0.021) but returned to preflight levels on R+4 (2.8% ± 2.9%; p = 0.959 Figure 3D). There was no change in carotid lumen IR (Figure 3C; p = 0.985). Although the carotid posterior wall intima IR value was not different from PRE values on FD150 (2.7% ± 3.0% PRE vs 7.1% ± 6.6% FD150; p = 0.137), the FD150 values were greater than those at both R+4 (1.4% ± 2.1%; p = 0.035) and R+6 m (0.8% ± 3.2%; p = 0.017) (Figure 3A p = 0.014).

## Discussion

The purpose of the current study was to use ultrasound imaging to evaluate the CC artery wall morphology and structure during long-duration spaceflight and post-spaceflight recovery. Results from this study demonstrate that long-duration spaceflight results in increased CC artery IMT and carotid wall IR, with the changes persisting till 4 days after returning to Earth but recovering after 6 months. This suggests that spaceflight results in vascular remodeling that is reversible upon returning to Earth.

Previous spaceflight studies have shown reduced velocity propagation time between the heart and the peripheral vascular area, suggesting increased vascular stiffness throughout the entire arterial system (Hughson et al., 2016; Baevsky et al., 2007). Similarly, another study found decreased CC artery distensibility after long-duration spaceflight, which was associated with altered responses to insulin (Hughson et al., 2016). Increased IMT was associated with increased posterior wall IR at 4 days postflight, which would suggest that spaceflight results in a remodeling process and/or a change in the carotid wall content, as suggested by previous studies (Arbeille et al., 2021; Arbeille et al., 2022).

The CC artery IMT close to the carotid bulb was increased inflight and 4 days after returning to Earth in the study. This is consistent with previous studies that have also shown increased carotid IMT with long-duration spaceflight (Arbeille and Provost, 2016; Arbeille et al., 2017b). However, in contrast to these findings, when the IMT was measured further away from the bulb (Figure 1), only a trend toward a greater value with spaceflight was found. A recent spaceflight study (Lee et al., 2020) found no significant change in carotid IMT with spaceflight; moreover, the amplitude of the IMT increase was found to be lower than that in our first 6-month inflight study (Arbeille and Provost, 2016). However, this could reflect a difference in the measurement location (Figure 1) from the point of the carotid bifurcation and the difference between the populations investigated. The location of the measuring point may impact the IMT value due to a gradual transition from a large elastic artery (middle CC) to a predominately muscular artery (bifurcation and internal carotid (Figure 1) (Kamenskiy et al., 2015) and indicate site-specific differences in adaptations and remodeling with spaceflight.

Neck muscle and carotid (middle part) posterior wall intima IR were moderately increased inflight but returned to preflight values 4 days after returning to Earth. The rapid recovery of muscle IR and posterior wall intima IR changes suggests that these adaptations are not related to a cellular modification but rather a transient change, such as microgravity-induced fluid shifts. The reduction of the jugular vein volume and the disappearance of the facial edema within 4 days following the return to normal gravity support such a hypothesis. Additionally, the lack of change in lumen IR at postflight day 4 might be in relation to the blood content (hemoglobin and hematocrit and density) changes or plasma volume alterations (Kunz et al., 2017).

In contrast to the rapid recovery of the posterior intima IR, the index of reflectivity of the carotid posterior wall was increased inflight and early postflight. This could be the result of either a cellular modification of the vessel wall structure or the inclusion of reflective particles, such as calcium, into the wall structure, as previously proposed (Arbeille et al., 2021; Arbeille et al., 2022). One may note that in both spaceflight and spaceflight analog studies, there is a significant loss of bone calcium that is released into the circulating blood along with a potential increase in circulating iron (Smith et al., 2015; Vico and Hargens, 2018; Nay et al., 2020). The incorporation of either calcium or iron into the vascular wall would serve to increase the reflective properties of the tissue, resulting in a greater IR value. Lower-limb arterial calcification with vascular aging (as measured by HR-pQCT) has been associated with abnormal bone microstructure and mineral density changes. Such findings suggest a possible pathophysiological link between osteoporosis and vascular calcification (Pacou et al., 2016). Other studies have suggested that vascular calcifications are actively regulated biological processes associated with the crystallization of hydroxyapatite in the extracellular matrix and in cells of the media or intima of the arterial wall (Lanzer et al., 2014). The carotid posterior wall IR normalization at 6 months postflight may be due (a) to the release of reflective particles that had penetrated the arterial wall or (b) to a reversible cellular modification that occurred. However, these two hypotheses still need to be clarified.

It has been reported that in patients with type 2 diabetes, medial sclerosis aggravates peripheral artery disease by promoting negative remodeling (Fok and Lanzer, 2018). Even if astronauts are not at risk (with hypertension hypercholesterolemia …), the increase in IMT should not be considered to be the starting point of future vascular diseases; however, if astronauts spend repeated long periods in space, they should undergo follow-up assessments of physical properties of the artery.

The changes in reflectivity and IMT reported in the present study are similar to those observed in diabetic patients and elderly persons (Gepner et al., 2014; Tesauro et al., 2017; Shirwany and Zou, 2010). Therefore, an alternative mechanism for the observed changes in carotid reflectivity and IMT could be related to the microRNA expression pattern (Malkani, 2020) and collagen and elastin transformations.

Although, on average, the carotid IMT and IR returned to preflight levels 6 months after returning to Earth, the values remained elevated for some of the astronauts. This could present an issue for longer-duration spaceflight and space exploration missions. Previous studies (Ray et al., 2015; Niamkey et al., 2021) of patients with atheromatous disease have reported that elevated CC IMT is associated with the long-term risk of developing first-ever carotid plaque, independent of traditional cardiovascular risk factors. Additionally, increased CC IMT contributed significantly, but modestly, to the incidence of cardiovascular disease (Kokubo et al., 2018; Tschiderer and Izzo, 2023). Therefore, the lack of recovery for some of the astronauts raises the questions of when or if the IMT and IR will return to the preflight values and whether these changes are indicative of cardiovascular disease development.

It should be noted that in the current study, astronauts with the sustained increase in IMT were not the same as those with the sustained increase in carotid wall IR. However, as the IMT is usually increased in patients with atherosclerosis and calcium is one of the major components of the atheromatous plaques, these two variables would be linked in the development of cardiovascular disease. Therefore, future spaceflight studies should assess the recovery of carotid IMT and carotid wall IR over longer periods of time to identify the parameters that might indicate the future development of cardiovascular disease.

As adaptations to the spaceflight environment may be precursors to disease development, appropriate countermeasures are required to ensure astronauts’ health. A recent study on a population of 14 subjects isolated in a large cavern with daily physical activity reported no change in carotid wall thickness, distensibility, or reflectivity (Arbeille et al., 2023). This suggests that the combined effect of gravity, daily exercise, and a lower stress environment is sufficient to protect the vasculature. Although astronauts perform periods of physical activity during spaceflight, additional countermeasures may still be needed to address adaptations to fluid shifts and the increased stress of the spaceflight environment, including physical unloading. A recent 60-day bedrest study reported that both exercise alone or combined exercise with artificial gravity had a protective effect on carotid wall IMT, whereas combined exercise and artificial gravity had a more significant effect on the carotid distensibility than exercise alone (Arbeille et al., 2024).

## Conclusion

The current study added to previous investigations that reported increased carotid artery stiffness and IMT during spaceflight and immediately following spaceflight (Hughson et al., 2016; Arbeille et al., 2017a) by showing that the carotid artery properties (IMT and posterior wall IR)were changed during spaceflight and at 4 days postflight but recovered by 6 months postflight. These findings suggest a reversible change in the structure (volume and remodeling) or content of the carotid wall.

In contrast, both neck muscle IR and posterior intima IR increased slightly with spaceflight but returned to preflight levels 4 days after returning to Earth, suggesting that the observed changes were the result of a more transient process, such as fluid redistribution with microgravity.

Although all variables returned to preflight levels 6 months after returning to Earth, sustained elevation in carotid IMT and posterior wall IR for some astronauts may be indicative of future cardiovascular disease development and require further study.

## Data Availability

The datasets presented in this article are not readily available due to the sensitive medical nature of this research. Requests to access the datasets should be directed to the corresponding author.
